# Understanding Social Support and Opinion Leaders in a Tuberculosis-Related Online Community in China: Content and Network Analyses

**DOI:** 10.2196/79140

**Published:** 2026-03-31

**Authors:** Xiaojun Fan, Jueman Zhang, Xiuli Wang

**Affiliations:** 1 School of New Media Peking University Beijing China; 2 Harrington School of Communication and Media University of Rhode Island Kingston, RI United States

**Keywords:** tuberculosis, TB, social support, online health community, OHC, social network analysis, semantic network analysis, opinion leader

## Abstract

**Background:**

Tuberculosis (TB) remains one of the world’s deadliest infectious diseases. Yet, despite the growing role of online health communities (OHCs) as key sources of social support, research on TB-related online communities remains scarce. Network analysis has been increasingly used to study OHCs and identify opinion leaders (OLs), offering a valuable approach to advancing knowledge about TB-related online communities.

**Objective:**

This study examined the types of social support and the influence of OLs in a prominent TB-related online forum in China, with a particular focus on its curated subforum that served as a centralized space for user interaction. The subforum consisted of posts recommended by the forum’s administrator and the corresponding user replies they generated.

**Methods:**

The data consisted of all 438 administrator-recommended posts and the 150,570 associated user replies over 18 years, from the forum’s launch in 2004 to 2021. The study used content analysis to examine the types of social support present in administrator-recommended posts, which are commonly considered high-quality. It then applied social network analysis to these posts and their associated user replies to identify OLs by using a Borda ranking method based on centrality measures and user tenure. Finally, semantic network analysis was used to explore topic clusters within each OL’s posts and their associated user replies.

**Results:**

The content analysis showed a high prevalence of informational and emotional support in the administrator-recommended posts. Of the 438 posts, 296 (67.5%) contained social support, with 150 containing informational support and 136 containing emotional support. Social support varied by post theme and whether the intent was to provide or seek it. Among disease knowledge posts, 74 out of 75 provided informational support. Emotional support was most frequently provided in nontreatment sharing posts (28/113) and most frequently sought in treatment experience posts (47/129). The social network analysis identified 10 OLs. The first was a former patient with TB, and the second was a pulmonary TB doctor. Together, they contributed 30.4% (133/438) of all the posts. Across the semantic network analyses of each OL’s posts and their associated user replies, informational support was more prominent than emotional support.

**Conclusions:**

The findings suggest that the examined TB-related online forum served as an important source of social support for people affected by TB in China, fostering an environment for both informational and emotional support. OLs played an important role by contributing posts and establishing a central position through reply interactions with users.

## Introduction

### Background

Tuberculosis (TB), caused by a bacterium called *Mycobacterium tuberculosis*, can be transmitted among people through the air and usually affects the lungs [1]. As of 2023, TB has likely reclaimed its position as the world’s leading cause of death from a single infectious agent after 3 years of COVID-19 leading, according to the World Health Organization [2]. Between 2014 and 2021, a total of 6,587,439 TB cases were reported in mainland China, with an average annual incidence rate of 59.17 per 100,000 people [3]. Although the TB incidence rate declined from 67.05 per 100,000 in 2014 to 46.40 per 100,000 in 2021 [3], China remains among the countries with the highest TB incidence rates, reporting about 734,400 new cases in 2023, ranking third globally and accounting for 6.8% of new cases worldwide [4].

TB treatment typically lasts at least 6 months. Throughout the treatment process, patients not only have to endure the physical pain caused by the disease and the side effects of medications but also experience negative impacts on their mental health [5-7]. Social support is considered a crucial factor in promoting treatment adherence, therapy completion, and the ability to navigate other challenges among patients with TB [8-10].

With the development of the internet and increased accessibility [11], online health communities (OHCs) have emerged as a leading source of social support for individuals facing health-related challenges, particularly through online forums [12,13] and social media groups [14-16]. The establishment and maintenance of a successful OHC requires key catalysts [17], often in the form of opinion leaders (OLs), who are defined as individuals in interpersonal communication networks that regularly provide information and opinions to others and exert influence on them [18]. Social network analysis has been increasingly used to identify influential users such as OLs and to reveal the community structure of online forums and social media groups [12-14].

Previous research has explored OHCs for various diseases [12-16]. However, the literature on TB-related OHCs remains scarce, which suggests a critical gap in understanding the state of online social support for people affected by TB. This study aimed to bridge this gap by exploring the TB forum in Baidu Post Bar (also known as Baidu Tieba, with “Bar” or “ba” meaning “forum”), a prominent TB-related OHC in China. Specifically, the study investigated the subforum within the TB forum that features posts recommended by the forum’s administrator, which are commonly regarded as high-quality, along with the corresponding user replies they generated. The data, consisting of the administrator-recommended posts and the associated user replies in the subforum, were extracted over a span of 18 years, from the TB forum’s launch in 2004 to 2021 [19].

The study has 3 main objectives. First, it uses content analysis to examine the types of social support provided and sought in the examined subforum. Second, it applies social network analysis to identify OLs using a Borda ranking method based on centrality measures and user tenure. Third, it uses semantic network analysis to uncover the topics discussed in the OLs’ posts and their associated user replies, offering insights into the types of social support they provided to users. To the best of our knowledge, this is the first study to apply social network analysis and semantic network analysis to a TB-related OHC. Understanding these aspects could enhance knowledge about TB-related online social support, inform the design of more effective digital health interventions, and help address gaps in TB education and support networks.

### Literature Review

#### Social Support in OHCs

The concept of social support involves providing information that makes individuals feel cared for, loved, valued, and connected within a network of reciprocal obligations [17]. It is postulated that social support positively impacts both physical and mental well-being [20], including alleviating stress and enhancing self-efficacy [17].

TB treatment generally requires at least 6 months, during which patients endure not only the physical discomfort caused by the disease and medication side effects, but also mental health challenges [5-7]. A study in China revealed that 59% of patients with TB experienced moderate to severe psychological stress [6]. A study from South Africa indicated that 60% of patients with TB exhibited symptoms of depression [7]. Social support is considered an important factor in helping patients with TB adhere to treatment, complete therapy, and overcome challenges [8-10]. A study from China demonstrated that social support from family members, friends, and other organizations contributed to improved medication adherence and alleviated symptoms of depression and anxiety [21].

Previous research outlines 4 primary types of social support: informational, emotional, instrumental, and appraisal support [22]. Another, more detailed classification includes informational, emotional, esteem, network, and tangible support [23,24]. A study on cancer-related OHCs identified opinion and personal narrative support as additional types in extending the detailed classification [12]. Furthermore, previous studies have revealed that informational and emotional support are the 2 most prevalent types of social support in OHCs for different health issues [25-27].

A scoping review of 49 studies worldwide about social support for people with TB and their households classified social support programs into 3 categories: financial intervention, food support, and community participation [8]. Among these, community participation fosters a supportive network and environment through activities such as providing educational resources and implementing educational activities [8]. However, limited access to these support programs remains a major challenge for many individuals [8].

As internet access continues to expand [11], OHCs have become a prominent source of social support for individuals facing health-related challenges, particularly through online forums [12,13] and social media platforms [14-16]. However, research on TB-related online social support remains limited, underscoring the need for further investigation into digital platforms to better understand the state of social support for patients with TB.

As a type of online community focused on health, OHCs share common characteristics such as social relationships among users, specific organizational structures and discussion formats, sharing of historical content, community rituals, and a shared online discussion space [28], all of which promote user identity, nurture long-term connections, and encourage sustained commitment to community goals [29].

Online communities enable users to initiate discussions and engage with others’ posts, fostering opportunities for interaction that help establish social networks [12]. In this context, online social support primarily refers to the support obtained through these interactions, often manifested in OHCs through the responses users receive from others [12]. Prior research has summarized that patients often turn to OHCs for social support due to limited access to adequate support in traditional networks, the convenience of computer-mediated communication, the need to cope with health-related stigma, and the perception of support providers as credible and similar [30].

According to Granovetter’s Weak Ties Theory [31], the interactive relationships within online communities are considered weak ties, which, unlike strong ties such as those with family and friends, often provide a wider range of information and have a lower likelihood of conflict [32]. Previous studies have revealed that OHCs facilitate patient self-management through the sharing of health information and experiences [29,33].

#### OLs in OHCs

Lazarsfeld et al [18] introduced the concept of OLs in the 2-step flow of communication theory, which suggests that media messages do not directly influence the public. Instead, information flows first from mass media to OLs—individuals who actively consume, interpret, and filter media content based on their knowledge and expertise. The OLs then disseminate and discuss the information within their social networks, shaping public opinions and behaviors [34,35]. Rogers’ [36] Diffusion of Innovation Theory also underscores the crucial role of OLs in spreading new information and influencing others. These individuals are typically influential figures within social networks, from whom others often seek information and advice [37].

Likewise, online OLs have been characterized as individuals who hold central positions in diffusing information within online communities and influencing public opinion [12,37]. A study on cancer-related OHCs revealed that online OLs actively shaped the agenda within OHCs by creating topics, primarily focusing on four themes: disease history and treatment, personal health and life updates, advocacy, and emotional expression [12].

In addition, research on cancer-related OHCs showed that online OLs provide various forms of social support through their replies to others, typically offering a combination of opinion support, emotional support, and network support [12]. However, a study on mental illness found that online OLs who post stereotypical and stigmatizing remarks on social media can reinforce public prejudices [38].

The influence of OLs in public health is evident not only in their role in disseminating health information but also in facilitating health promotion programs, including serving as role models for behavior change [39].

Within online communities, OLs have significantly more connections and a greater ability to foster shared attitudes among community members [12,40]. With the widespread use of mobile communication, online OLs can deliver information and advice to members within OHCs more promptly and effectively than those within traditional offline social networks [41].

Regarding the identity of OLs in OHCs, one study on cancer-related OHCs found that individuals with higher levels of cancer knowledge and a more optimistic outlook on life challenges were more likely to become OLs in these communities [42]. Another study on cancer-related OHCs identified the majority of OLs as patients with cancer, while the remaining were family or friend caregivers [12].

#### Research Questions

This study examined the subforum within the TB forum in Baidu Post Bar that features administrator-recommended posts, along with the replies those posts received, collected over 18 years from the TB forum’s launch in 2004 to 2021. To understand the types of social support in the administrator-recommended posts, we used a 2-step content analysis. First, we classified each of the posts into 1 theme, drawing on existing classifications from the literature on OHCs [12,13], as well as emerging ones observed in the posts. The 6 resulting themes were disease knowledge, treatment experience, nontreatment sharing, community activities, community announcements, and questions and answers. Then, we identified posts containing social support and categorized them into 1 of 4 types based on the literature: informational, emotional, instrumental, or network [12,22]. We further distinguished whether each post provided or sought support. We posed the following questions:

- Research question 1 (RQ1): What types of social support are provided and sought in the administrator-recommended posts?
- Research question 2 (RQ2): How do different types of social support vary by post theme?

In addition, we used social network analysis to identify OLs, assessed by centrality and user-tenure metrics. We also applied semantic network analysis to uncover the topics discussed in the OLs’ posts and their associated user replies. We posed the following questions:

- Research question 3 (RQ3): Who are the opinion leaders in the subforum based on centrality and user-tenure metrics?
- Research question 4 (RQ4): What topics emerge from opinion leaders’ posts and their associated user replies?

## Methods

### Data Source

We examined the TB forum hosted on Baidu Post Bar, one of China’s most prominent online communities related to TB. Baidu Post Bar, also known as Baidu Tieba, was launched in 2003 and represents one of the earliest and largest interest-based online platforms in China. Organized around thematic “bars” (forums) dedicated to specific topics, Baidu Post Bar enables registered users to initiate discussion threads or participate in existing ones through replies. At its peak around 2015, Baidu Post Bar encompassed over 22 million forums and attracted approximately 300 million monthly active users. Although subsequent competition from emerging social media platforms has reduced its dominance [19,43], Baidu Post Bar remains a valuable case for studying Chinese internet culture and community dynamics, owing to its vast historical user base and distinctive user-generated content ecosystem. The TB forum, as one of the platform’s earliest and most active disease-specific communities, offers a unique dataset for investigating the core mechanisms driving health-related online communities—namely, social support and opinion leadership.

We focused our analysis on the subforum of administrator-recommended posts within the TB forum. In Baidu Post Bar, an administrator holds the highest authority and is responsible for forum governance, policy enforcement, and user management. Each forum includes a prominent navigation tab leading to a centralized subforum for all administrator-recommended posts, which are widely perceived as high-quality content. This subforum fosters focused user engagement through replies, which are the sole interaction mechanism. In contrast, the broader forum contains numerous low-engagement threads, yielding fragmented reply networks unsuitable for robust social network analysis. By restricting the dataset to the subforum containing administrator-recommended posts and their associated user replies, we ensured a cohesive, high-interaction dataset that was optimal for examining social support and opinion leadership dynamics.

Data collection spanned from 2004 to December 31, 2021, encompassing the TB forum’s entire lifecycle—from its inception in 2004 through Baidu Post Bar’s peak popularity and its subsequent decline amid competition from newer social media. This longitudinal window captures the forum’s rise, maturity, and waning activity, culminating in the near cessation of administrator-recommended posts by late 2021 (only 2 posts in 2021) and a registered user base of approximately 113,000. Using Scrapy [44], an open-source web scraping framework for Python (Guido van Rossum), we programmatically retrieved 438 original administrator-recommended posts and 150,570 associated user replies, with the earliest post dated May 27, 2004. Additional publicly available metadata included post and reply timestamps, anonymized user identities, gender, registration date, and lifetime post counts.

### Content Analysis to Examine Themes and Social Support in the Posts

Content analysis has often been used to examine social support in OHCs [24,25,45]. The unit of analysis is an original post in the administrator-recommended subforum. Each post consists of a user identity, post date, post title, and post body. We used a 2-step content analysis to manually code each post’s theme and social support based on its title and body. In step 1, the researchers classified each of the 438 posts into 1 of 6 themes, drawing on existing classifications from the literature on OHCs [12,13], as well as emerging ones observed in the posts. The first theme, disease knowledge, includes information about TB treatment, research progress, and advice on returning to work or school. The second theme, treatment experience, consists of patients sharing their experience with TB treatment. The third theme, nontreatment sharing, covers aspects of daily life or travel experience during or after TB treatment, as well as personal talents and skills. The fourth theme, community activities, contains information about group activities initiated within the OHC, including offline events. The fifth theme, community announcements, consists of updates shared by the management team of the TB OHC. The final theme, questions and answers, covers consultations on TB-related issues and responses provided by doctors or experienced patients.

In the second step, the researchers identified 296 of the 438 posts as containing social support. These 296 posts were then classified into 1 of 4 types of social support—informational, emotional, instrumental, and network support—based on prior literature [12,22]. In addition, each post was coded according to whether it provided or sought social support. Informational support includes advice, knowledge, and resources related to treatment options, medication use, hospital information, research findings, and other relevant topics to help individuals navigate TB-related challenges and make informed decisions. Emotional support involves expressing empathy, concern, compassion, and encouragement to help individuals cope with TB-related struggles and difficult emotions. Instrumental support refers to practical resources and assistance to help individuals address their needs. Network support focuses on fostering a sense of belonging within a community through actions such as offering greetings, acknowledging other group members, and engaging in mutual information exchange.

For example, a post titled “Is there anyone familiar with bronchial TB? What medications are you taking?” (including its body text) was coded under the disease knowledge theme as a request for informational support. Another post titled “I’m not afraid of anything, except for making my parents worry” (including its body text) was coded under the treatment experience theme as seeking emotional support. Two researchers independently coded 50 posts randomly selected from the 438 posts to test intercoder reliability. Krippendorff’s α was 0.92 for classifying post themes, 0.89 for classifying the type of social support a post provides, and 0.90 for classifying the type of social support a post seeks. Each researcher then coded half of the remaining posts independently.

### Social Network Analysis to Examine Community Structure and Identify OLs

This study used social network analysis to examine the community structure and to identify OLs within the subforum featuring administrator-recommended posts and their associated user replies. Social network analysis involves analyzing the structure of a social network, which consists of a set of actors connected by 1 or more relationships. In this context, nodes represent users, and edges represent their connections [46]. We modeled the subforum as a directed interaction network, where each node represents a user and a directed edge from user *i* to user *j* indicates that user *i* replied to user *j*, which is the only form of interaction available in the subforum.

We identified the top 10 OLs using a Borda ranking method based on 4 centrality metrics and a user-tenure metric [47,48]. User tenure was included as a separate indicator in the Borda aggregation to capture cumulative participation in the forum, rather than to normalize or adjust the structural centrality measures. The 4 centrality metrics are (1) normalized in-degree centrality (the proportion of distinct users who replied to a given user); (2) normalized out-degree centrality (the proportion of distinct users a given user replied to); (3) betweenness centrality (the extent to which a user lies on shortest reply paths between other users); and (4) PageRank (a weighted measure that reflects replies from other highly influential users) [40]. User tenure was operationalized as the time since registration. Each metric was first converted into a separate rank, and these ranks were then aggregated using a Borda count to produce an overall influence score as follows.

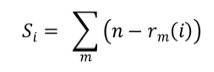



where *r_m_(i)* is the rank of user *i* on metric *m* among *n* users. The final Borda rank was derived from ordering the users by S_i_.

Gephi 0.10.1 (Mathieu Bastian, Sébastien Heymann, and Mathieu Jacomy) was used to conduct the social network analysis [49], using the Yifan Hu proportional layout algorithm.

### Semantic Network Analysis of OLs’ Posts and All User Replies

This study used semantic network analysis to investigate the topics discussed in OLs’ posts and user replies. Semantic network analysis is frequently used to analyze large volumes of text and identify the main topics. Unlike social network analysis, which treats actors as nodes and relationships as edges, semantic network analysis treats words as nodes and their co-occurrences as edges [46]. In a semantic network, words that convey the same topic tend to appear together in the same cluster. The higher the frequency with which a specific group of words appears in the semantic network, the more prominent the corresponding topic.

Since the corpus used in this study was in Chinese, we used ROST-CM6 (Wuhan University), a commonly used Chinese content mining and analysis software. Initially, all texts were tokenized, and high-frequency words were extracted based on a frequency ranking of the top 200 words. Subsequently, high-frequency words with similar meanings were merged. Word pairs exhibiting co-occurrence relationships within the set of high-frequency words were then used to construct a co-occurrence matrix. Finally, the semantic networks of high-frequency words were visualized using Gephi (Mathieu Bastian, Sébastien Heymann, and Mathieu Jacomy) [47], and the words were translated from Chinese into English for presentation.

### Ethical Considerations

This study analyzes publicly available, anonymized data obtained from an online platform. No private, personally identifiable, or sensitive information was accessed or collected. All data were deidentified before analysis. The research did not involve direct interaction with human participants. In accordance with items (1) and (2) of Article 32 of the Measures for Ethical Review of Life Science and Medical Research Involving Human Subjects, jointly issued by the National Health Commission, the Ministry of Education, the Ministry of Science and Technology, and the National Administration of Traditional Chinese Medicine of the People’s Republic of China, the study was exempt from institutional review board review for the following reasons. The research is conducted using legally obtained public online data generated through observation without interfering with public behaviors; all data have been anonymized; the study causes no harm to the human body and does not involve sensitive personal information or commercial interests [50].

## Results

### Original Posts by Year

**Figure 1:**
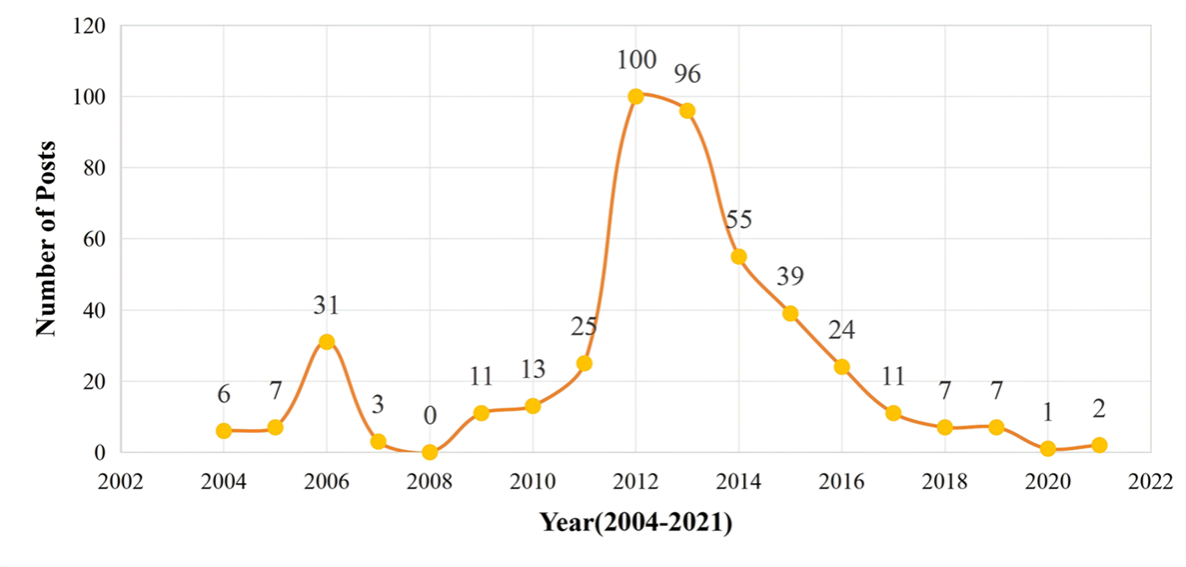
The number of administrator-recommended posts from 2004 to 2021.

Figure 1 illustrates the annual number of original posts in the TB forum from its launch in 2004 through 2021. The peak occurred in 2012, followed closely by 2013. This surge can be attributed primarily to 2 factors. First, the 2012-2013 period coincided with the height of user activity on Baidu Post Bar, which drew a large number of patients with TB to the forum for interaction and support. Second, the production of high-quality posts depended heavily on active moderation and recommendation by the forum administrator. In 2012, a new administrator took over the role. Compared to the previous one, the new administrator exhibited greater engagement, stronger organizational skills, and more substantial influence within the community. He authored 24 posts in 2012 and 15 in 2013, while recommending 140 posts over those 2 years.

Subsequently, the conditions supporting sustained content production eroded. Core users gradually left the forum upon recovery, and more significantly, the emergence of new social media platforms such as Weibo (known as China’s Twitter; Sina Corporation) and WeChat (a super app developed by Tencent and widely used for messaging, social networking, mobile payments, and various online services) prompted a broader migration away from Baidu Post Bar. As a result, the volume of administrator-recommended posts declined steadily over the year. By 2020 and 2021, the number of administrator-recommended posts had fallen to just 1 and 2 posts, respectively.

### Themes and Social Support in Administrator-Recommended Posts

Table 1 presents the classification of themes and social support in the 438 original posts. Among the 6 themes, the most prevalent was treatment experience, where patients shared their experience with TB treatment, appearing in 29.5% (129/438) of the posts. This was followed by nontreatment sharing at 25.8% (113/438), disease knowledge at 17.1% (75/438), community activities at 16% (10/438), community announcements at 7.5% (33/438), and questions and answers at 4.1% (18/438).

**Table 1:** Classification of themes and social support in administrator-recommended posts (N=438). One example post title (labeled a-i) is provided for each category containing ≥10 posts. Coding was based on both the post title and the body text.

Theme	Posts (N=438), n (%)	Social support (n=296), n (%)	Types of social support									
			Informational (n=150), n (%)		Emotional (n=136), n (%)			Instrumental (n=4), n (%)			Network (n=6), n (%)	
			Provide	Seek	Provide	Seek	Provide		Seek	Provide		Seek
Disease knowledge	75 (17.1)	75 (25.3)	74^a^ (16.9)	0 (0)	1 (0.2)	0 (0)	0 (0)		0 (0)	0 (0)		0 (0)
Treatment experience	129 (29.5)	115 (38.9)	25^b^ (5.7)	12^c^ (2.7)	30^d^ (6.8)	47^e^ (10.7)	0 (0)		1 (0.2)	0 (0)		0 (0)
Nontreatment sharing	113 (25.8)	41 (13.9)	3 (0.7)	1 (0.2)	28^f^ (6.4)	8 (1.8)	0 (0)		0 (0)	1 (0.2)		0 (0)
Community activities	70 (16)	29 (9.8)	3 (0.7)	2 (0.5)	18^g^ (4.1)	4 (0.9)	1 (0.2)		1 (0.2)	0 (0)		0 (0)
Community announcements	33 (7.5)	18 (6.1)	12^h^ (2.7)	0 (0)	0 (0)	0 (0)	1 (0.2)		0 (0)	5 (1.1)		0 (0)
Questions and answers	18 (4.1)	18 (6.1)	12^i^ (2.7)	6 (1.4)	0 (0)	0 (0)	0 (0)		0 (0)	0 (0)		0 (0)
Total	438 (100)	296 (67.5)	129 (29.5)	21 (4.8)	77 (17.6)	59 (13.5)	2 (0.5)		2 (0.5)	6 (1.4)		0 (0)

^a^Example post title: is there anyone familiar with bronchial tuberculosis? What medications are you taking?

^b^Example post title: process record: treatment of tuberculosis in Canada.

^c^Example post title: record my first day on medication.

^d^Example post title: to those who have recovered and finished medication, check in here and cheer on the newcomers.

^e^Example post title: I am not afraid of anything, except for making my parents worry.

^f^Example post title: [positive energy] teenager with pulmonary tuberculosis dances the shuffle and shows that patients with tuberculosis are no different from others!

^g^Example post title: dear friends, whether we know each other or not, let’s comfort and encourage one another!

^h^Example post title: hello everyone! To help you better communicate, interact, and seek advice in this forum, please read this post carefully. It will answer common questions and explain the forum’s rules. We hope this guide is helpful to you!

^i^Example post title: [back-to-school-season] questions and answers on updated conditions for suspension and resumption of studies—all students welcome!

RQ1 examined the types of social support provided and sought in the subforum featuring administrator-recommended posts. As Table 1 shows, about 67.5% (296/438) of the posts contained social support. Among the 4 types of social support, informational support was the most prevalent, appearing in 150 posts, with 86% (129/150) providing it and 14% (21/150) seeking it. This was closely followed by emotional support, present in 136 posts, with 56.6% (77/136) providing it and 43.4% (59/136) seeking it. Instrumental support and network support were minimal, found in 4 and 6 posts, respectively.

RQ2 explored how different types of social support varied across post themes. Analysis of the distribution of informational and emotional support across the 3 most common themes—treatment experience, nontreatment sharing, and disease knowledge—revealed distinct patterns. Among the 129 posts about treatment experience, 89.1% (115/129) contained social support. Seeking emotional support was the most prevalent, appearing in 40.9% (47/115) of the posts, followed by 26.1% (30/115) posts providing emotional support, 21.7% (25/115) providing informational support, and 10.4% (12/115) seeking informational support. In contrast, only 36.3% (41/113) of posts about nontreatment sharing included social support, with providing emotional support being the most dominant type (68.3%, 28/41 of the posts). For the 75 posts about disease knowledge, providing informational support was overwhelmingly dominant, appearing in 98.7% (74/75) of posts. Table 2 presents the top 10 most-replied-to posts that contain social support.

**Table 2:** Top 10 most-replied-to posts containing social support.

Reply rank	Reply count	Post translated from Mandarin^a^ (date posted)	Theme coded in the content analysis	Social support type was coded in the content analysis
1	37,474	You’re welcome to ask the doctor about TB^b^ (Feb 6, 2006)^c^.	Treatment experience	Informational (provide)
2	30,075	Record my first day on medication (Sept 17, 2011).	Treatment experience	Informational (seek)
3	7844	A hub for comforting communication (Oct 4, 2011).	Community activities	Emotional (seek)
4	7395	I’m not afraid of anything, except for making my parents worry (Aug 23, 2011).	Treatment experience	Emotional (seek)
5	7217	I’ll live well (Nov 6, 2012).	Non-treatment sharing	Emotional (seek)
6	7057	July 2015: Girls who love to smile have good luck (Feb 24, 2016).	Non-treatment sharing	Emotional (provide)
7	6436	Anti-TB Diary (Sept 2, 2014).	Treatment experience	Informational (provide)
8	6332	Process record: treatment of TB in Canada (Aug 15, 2014).	Treatment experience	Informational (provide)
9	6217	Recording daily medication with photos and text—Stay persistent and keep going! (July 10, 2013)	Treatment experience	Emotional (provide)
10	5779	Process record: treatment of TB in Canada (Apr 5, 2015).	Treatment experience	Informational (provide)

^a^Each original post was manually coded for theme and social support based on its title and body. Due to space limitations, the table presents only the post titles rather than the full post content.

^b^TB: tuberculosis.

^c^The user who posted the post is a doctor specializing in pulmonary tuberculosis.

### Identifying OLs

**Figure 2:**
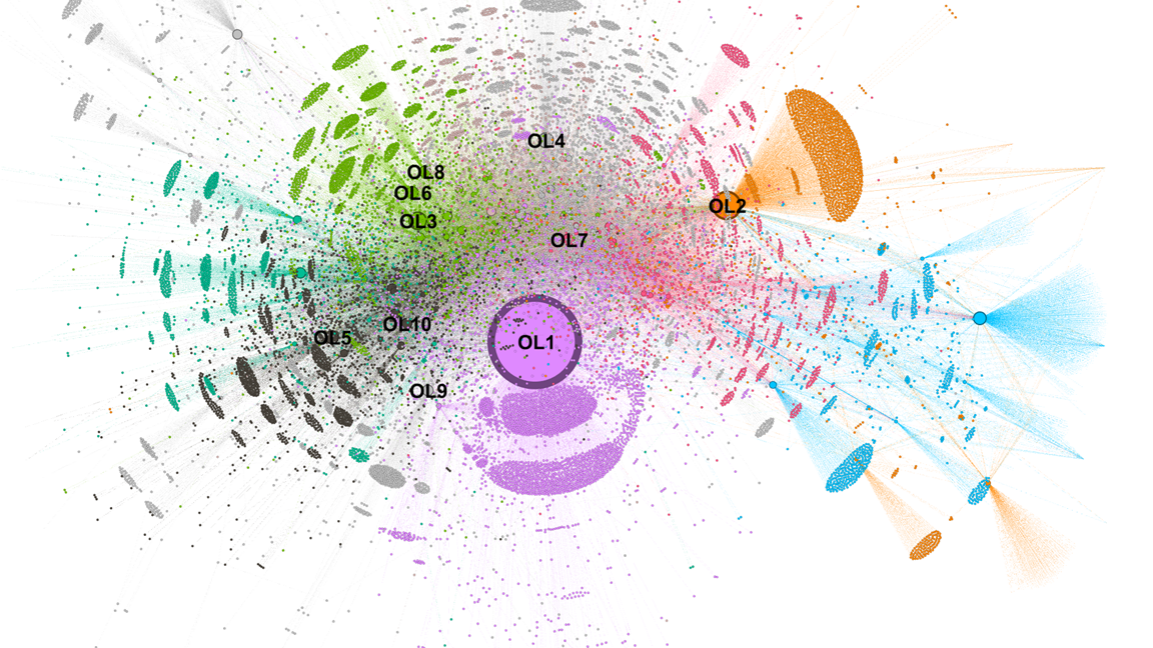
Opinion leaders (OLs) and subcommunities of the administrator-recommended posts and user replies.

Figure 2 depicts the overall reply-based social network within the TB subforum of administrator-recommended posts, clustered using the modularity module via the Louvain algorithm and visualized with the Yifan Hu proportional layout algorithm in Gephi [47]. The network reveals a highly centralized structure dominated by the top 10 OLs (labeled OL1-OL10), each surrounded by dense clusters of varying colors representing distinct interaction communities. OL1 (purple) commands the largest and most cohesive cluster, indicating paramount influence and extensive reply engagement. Other leaders—such as OL2 (orange), OL3 (green), and OL7 (pink)—anchor substantial but smaller subnetworks, while peripheral clusters exhibit sparser connections. This configuration underscores the gatekeeping role of OLs, channeling the majority of discourse and support exchange, consistent with a hierarchical, star-like topology characteristic of health-related online communities where influential users mediate information flow and relational ties.

Table 3 presents the top 10 OLs identified using the Borda ranking method, which aggregates scores across 4 centrality metrics and a user-tenure metric.

**Table 3:** Top 10 opinion leaders identified using the Borda ranking method.

User ID^a^	Rank^b^	Indegree centrality (normalized)	Outdegree centrality (normalized)	Betweenness centrality	PageRank	User tenure (years)
OL^c^1	1	0.2215	0.1262	0.2368	0.0599	14.4
OL2	2	0.0920	0.0103	0.0441	0.0206	16.9
OL3	3	0.0311	0.0301	0.0275	0.0072	6.7
OL4	4	0.0249	0.0195	0.0201	0.0073	5.8
OL5	5 (tie)	0.0473	0.0048	0.0136	0.0097	7.9
OL6	5 (tie)	0.0252	0.0137	0.0140	0.0061	9.3
OL7	7	0.0256	0.0126	0.0132	0.0066	9.3
OL8	8	0.0179	0.0168	0.0174	0.0061	8.4
OL9	9	0.0214	0.0180	0.0153	0.0050	7.2
OL10	10	0.0181	0.0164	0.0122	0.0042	7.8

^a^ID: identity.

^b^For the Borda ranking, each metric was converted into ranks separately. These ranks were then combined using a Borda count aggregation method, as explained in the Methods section.

^c^OL: opinion leader.

The top-ranked OL led all 4 centrality measures: receiving replies from the largest proportion of distinct users (highest normalized in-degree centrality), replying to the largest proportion of distinct users (highest normalized out-degree centrality), occupying the most structurally important positions in reply paths (highest betweenness centrality), and receiving replies from other highly influential users (highest PageRank). Although this user did not have the longest tenure in the forum—the second-ranked OL had been active for more years—the consistently superior centrality scores outweighed the tenure difference in the Borda aggregation, resulting in this user’s overall first-place ranking.

The first-ranked OL served as the administrator of the TB forum at the time of data collection and is a former patient with TB who had completed treatment and fully recovered. The second-ranked OL, a medical doctor specializing in pulmonary TB, is the founder and initial administrator of the TB forum.

Out of the 438 administrators-recommended posts, the first OL contributed 71 posts, accounting for 16.2% of the total. The second OL contributed 62 posts, accounting for 14.2% of the total. Combined, the 2 OLs contributed 30.4% (133/438) of the posts.

### Semantic Network Visualization of Topics in OLs’ Posts and User Replies

**Figure 3:**
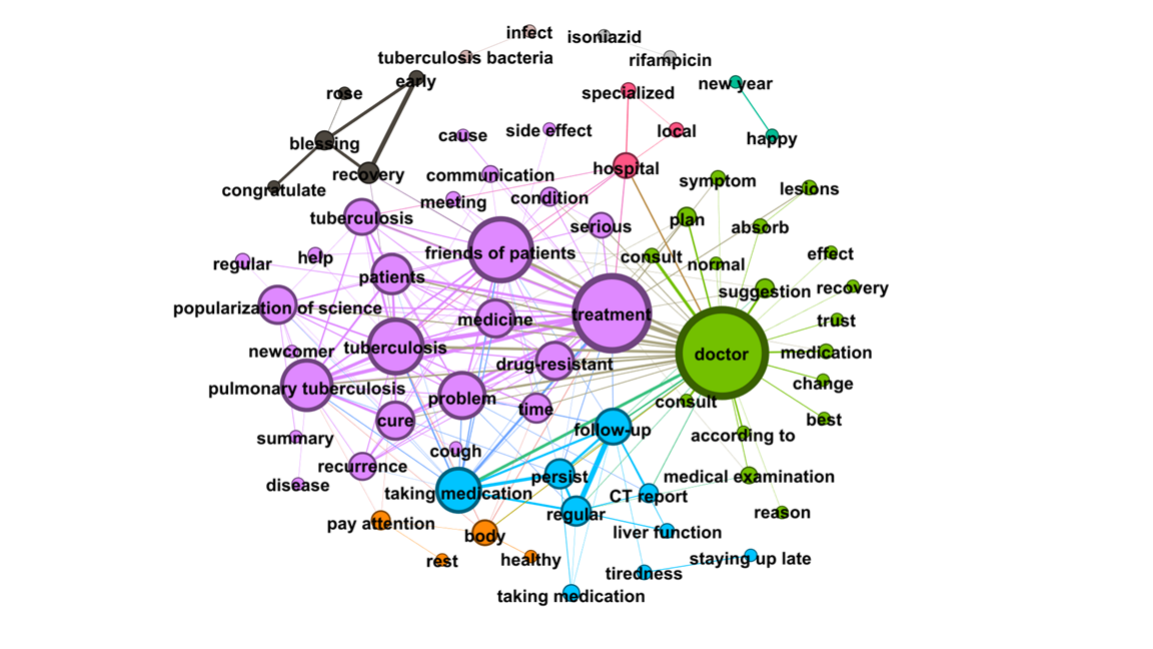
Semantic network of opinion leader 1 (OL1) posts and associated user replies.

**Figure 4:**
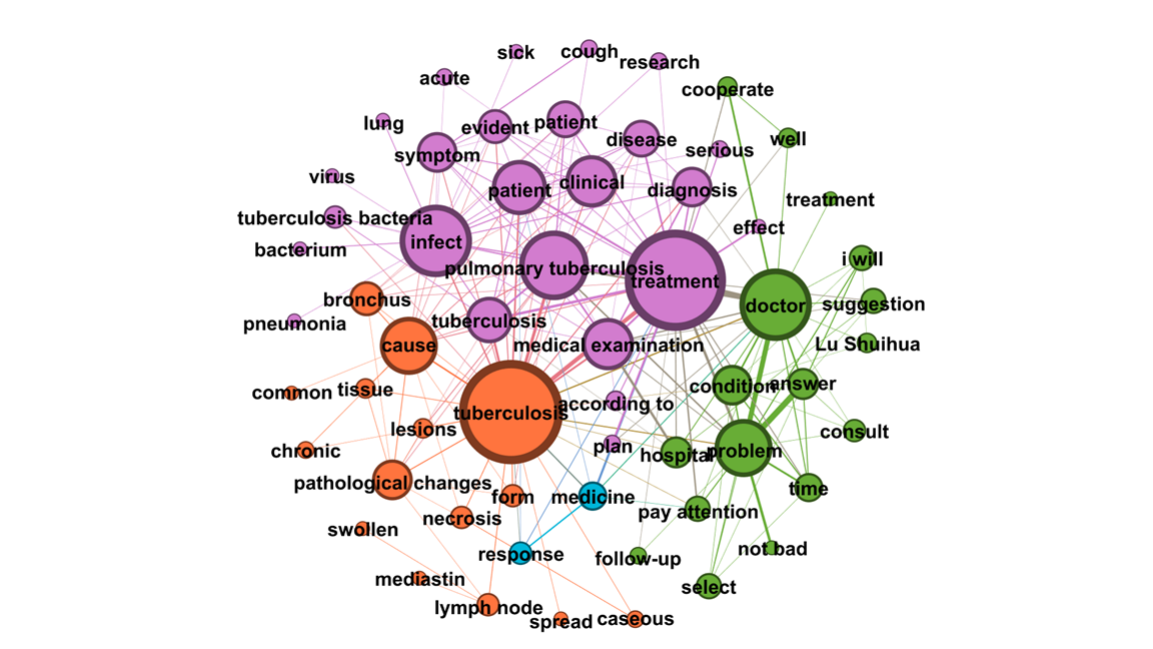
Semantic networks of opinion leader 2 (OL2) posts and associated user replies.

**Figure 5:**
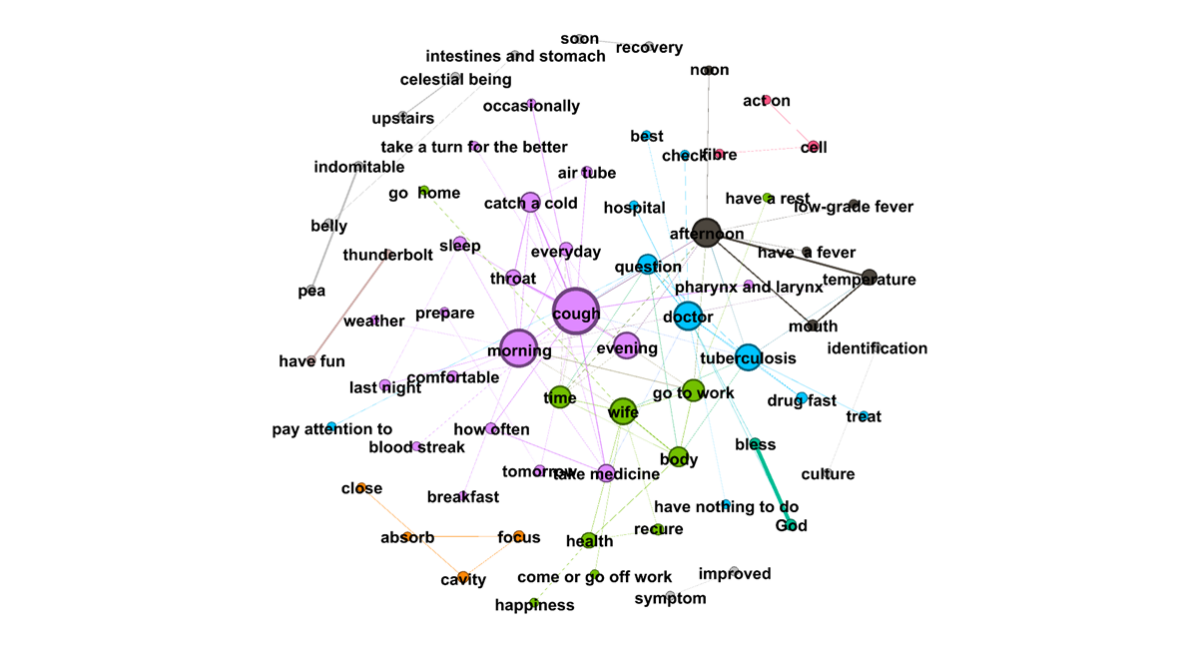
Semantic networks of opinion leader 3 (OL3) posts and associated user replies.

**Figure 6:**
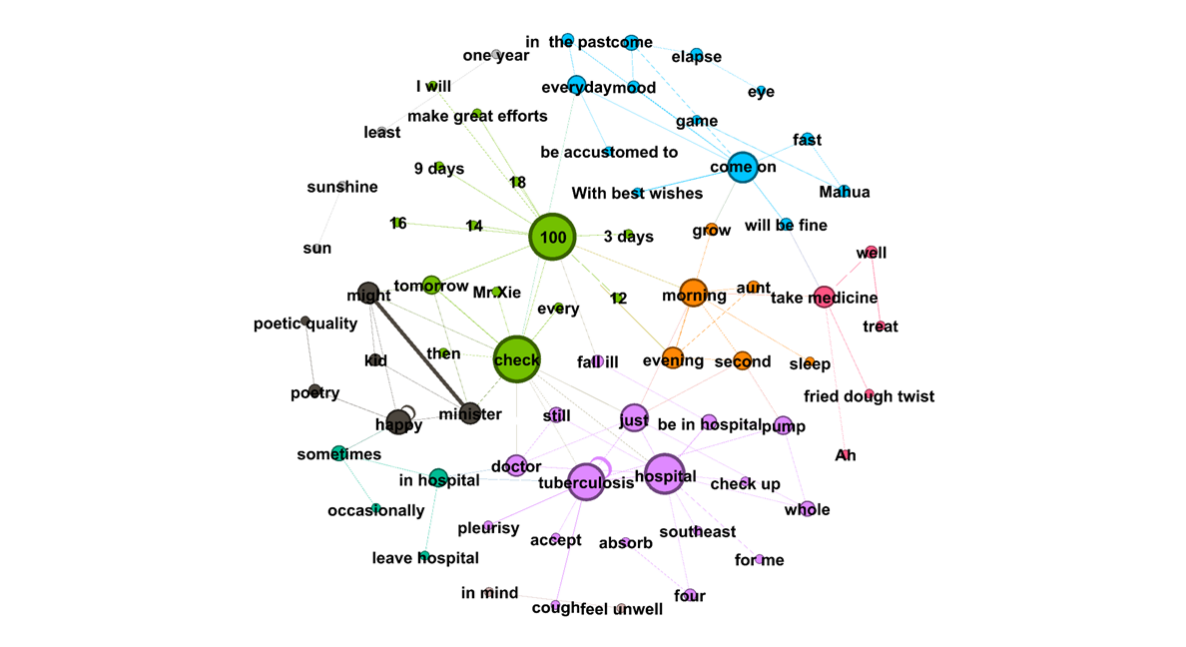
Semantic networks of opinion leader 4 (OL4) posts and associated user replies.

**Figure 7:**
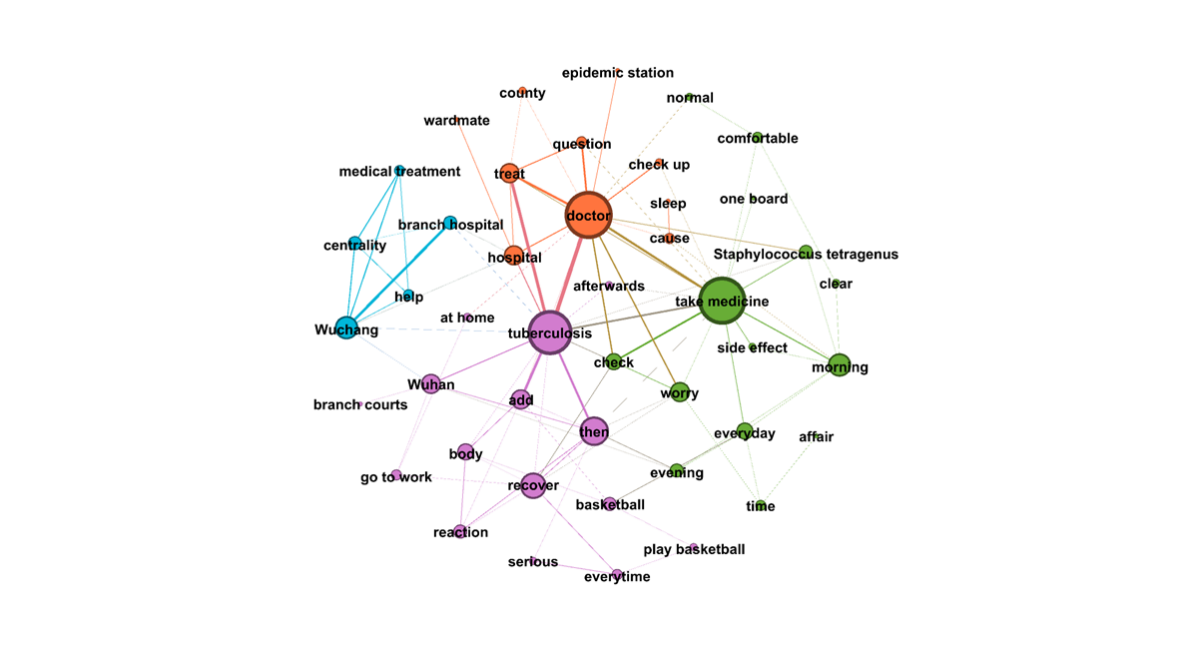
Semantic networks of opinion leader 5 (OL5) posts and associated user replies.

**Figure 8:**
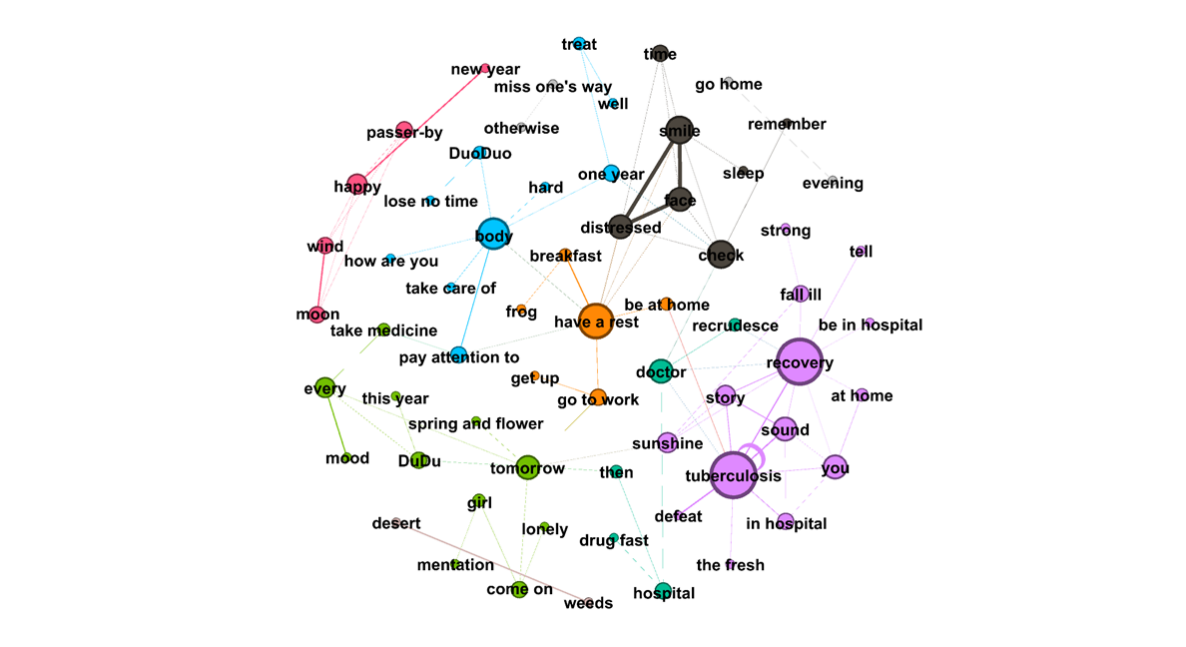
Semantic networks of opinion leader 6 (OL6) posts and associated user replies.

**Figure 9:**
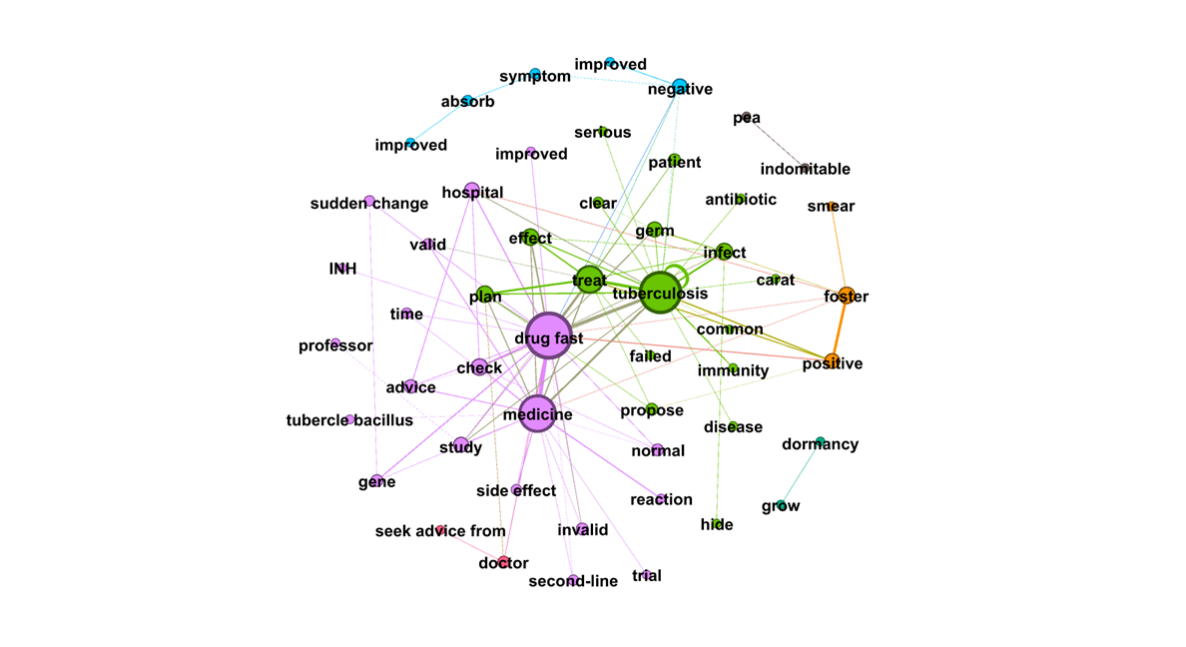
Semantic networks of opinion leader 7 (OL7) posts and associated user replies.

**Figure 10:**
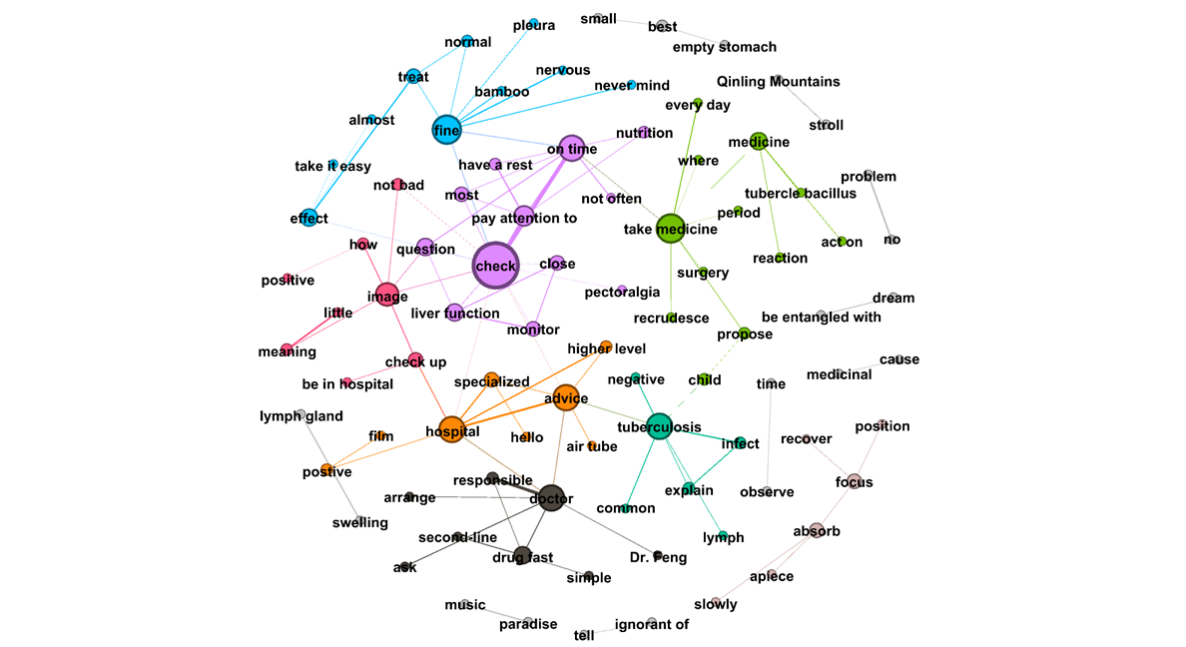
Semantic networks of opinion leader 8 (OL8) posts and associated user replies.

**Figure 11:**
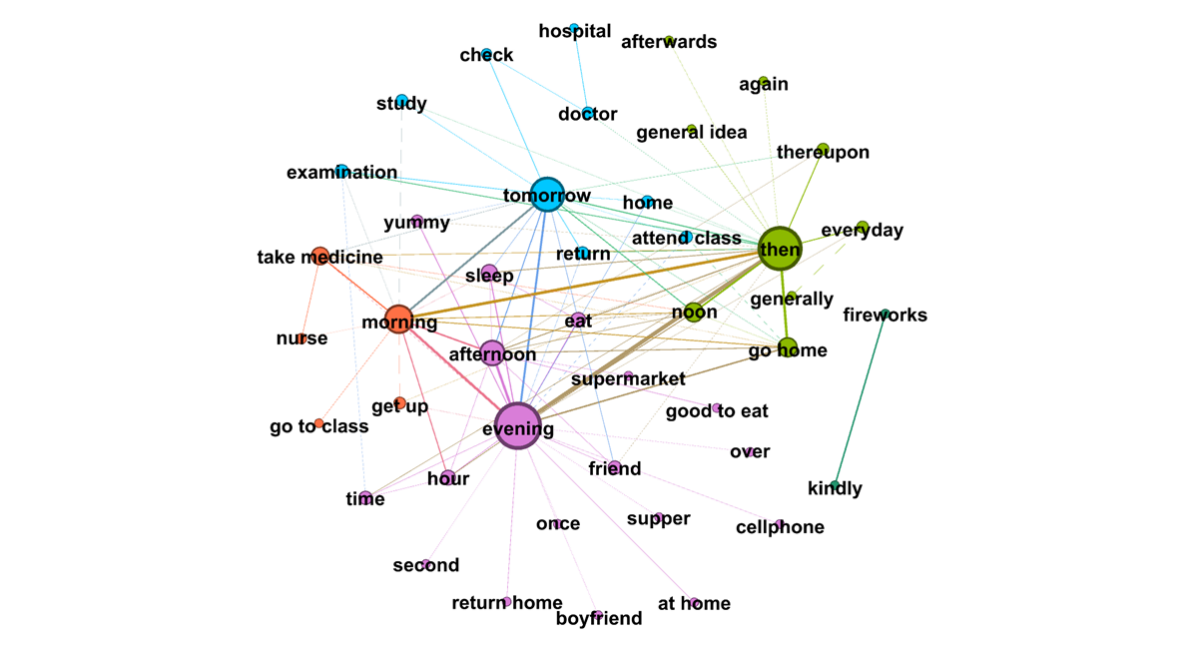
Semantic networks of opinion leader 9 (OL9) posts and associated user replies.

**Figure 12:**
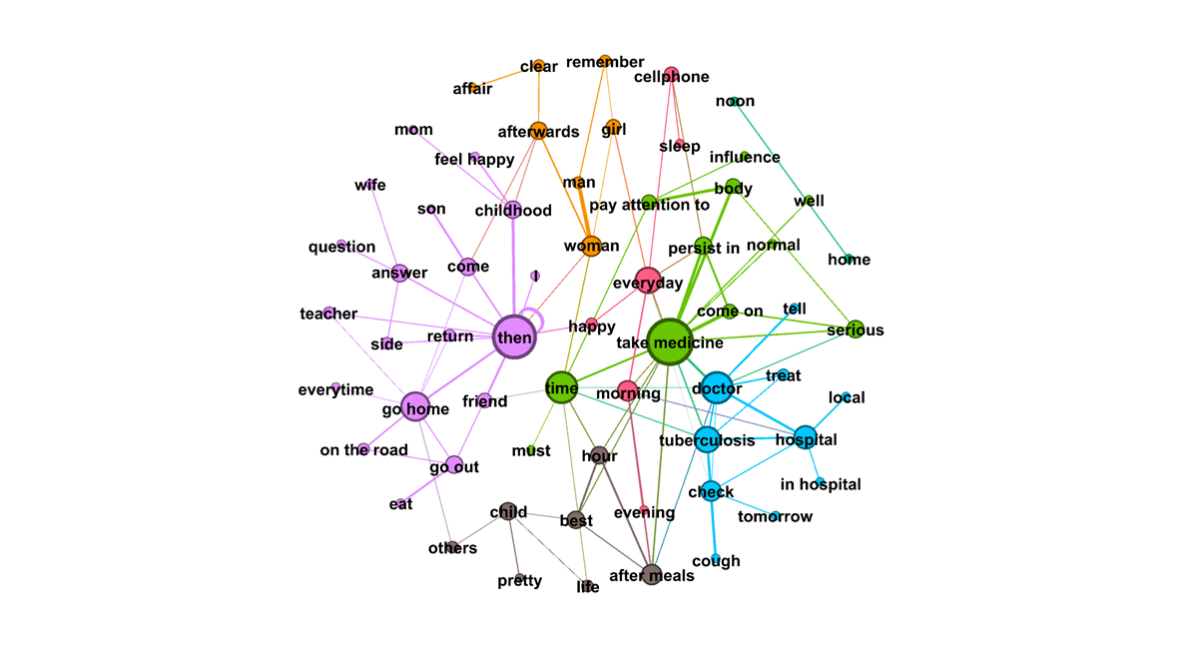
Semantic networks of opinion leader 10 (OL10) posts and associated user replies.

RQ4 examined the topics that emerged from OLs’ posts and the replies these posts received from users. Semantic network visualization, achieved using the modularity module via the Louvain algorithm, was used to uncover the topics emerging from each of the top 10 OLs’ posts and their associated replies. Figures 3-12 present the resulting semantic networks, with Figure 3 corresponding to OL 1, Figure 4 to OL 2, and so forth to Figure 12. In these visualizations, each node represents a high-frequency word translated from Chinese, with node size proportional to its frequency of co-occurrence with other high-frequency words. Edge thickness signifies the strength of co-occurrence between word pairs. Colors delineate distinct topic clusters but may not reflect cluster magnitude in terms of the number of words or nodes within each cluster.

As illustrated in Figure 3, the semantic network constructed from the first OL's posts and the associated user replies identifies seven distinct topic clusters, each denoted by a unique color and characterized by its high-frequency words. The largest cluster (purple, n=25 words) focuses on TB treatment information, including transmission routes, therapeutic options for contacts, pulmonary TB, drug resistance, cure rates, recurrence risks, and treatment duration. The second-largest cluster (green, n=18 words) revolves around the keyword “doctor,” emphasizing the importance of professional medical consultation and treatment. The third cluster (blue, n=9 words) addresses treatment adherence and lifestyle guidelines, including regular check-ups, medication compliance, liver protection, and avoidance of overexertion. The fourth cluster (black, n=5 words) conveys blessings and wishes for speedy recovery. The fifth (orange, n=4 words) highlights rest and overall health maintenance. The sixth (gray, n=4 words) pertains to antibiotic regimens for TB. Finally, the smallest cluster (teal, n=2 words) reflects New Year greetings.

The first OL exemplifies both informational and emotional support in interactions with forum users. For instance, when a user inquired whether joint pain during medication treatment was normal, the first OL responded: “Joint pain is considered normal; many patients experience it after taking the medication. Check your liver and kidney function—if it’s caused by high uric acid, your attending doctor can provide appropriate treatment.” This exchange illustrates informational support through practical medical guidance and reassurance based on clinical knowledge.

Emotional support is equally evident. When responding to a distressed minor patient, the first OL wrote: “Tuberculosis is no longer a terminal illness. It’s normal to feel shocked and panicked at first due to a lack of understanding. With proper treatment and adherence to medication, it can be cured. Many patients in this forum have already recovered, and you will too.” Such replies offer empathy, normalization of fear, and hopeful encouragement, fostering resilience amid stigma and uncertainty.

Notably, the first OL’s most engaged posts—generating 3693 replies—explicitly called on recovered patients with TB to share encouragement with newly diagnosed individuals, reinforcing the forum’s role as a mutual-aid network driven by peer expertise and solidarity. These examples underscore how OLs blend authoritative knowledge with compassionate outreach, enhancing both cognitive understanding and affective coping in the community.

Figure 4 depicts the semantic network derived from the second OL’s posts and the associated user replies. The visualization identifies 4 topic clusters, each delineated by a distinct color and characterized by its high-frequency words. The largest cluster (purple, n=25 words) centers on TB symptoms, diagnosis, and treatment, with particular emphasis on pulmonary TB. The second-largest cluster (green, n=17 words) revolves around the keyword “doctor,” underscoring the importance of seeking advice and treatment from doctors. The third cluster (orange, n=15 words), anchored by the keyword “TB,” highlights patient symptoms and clinical characteristics, featuring words such as “bronchus,” “pathological changes,” “lesions,” and “lymph nodes.” The smallest cluster (blue, n=2 words) comprises “medicine” and “response,” reflecting discussions of pharmacological reactions or treatment responses.

Notably, the second OL’s 2006 post titled “Ask the Doctor” received an extraordinary 37,474 replies, making it the most-replied-to post in the subforum. For instance, 1 user asked whether rifampin and other anti-TB drugs could be taken after meals. The second OL responded: “Rifampin should be taken on an empty stomach, while other medications do not have such strict requirements. This is determined by the drug’s mechanism of action.” Such interactions highlight the second OL’s pivotal role in the community’s formative years, delivering professional consultations that bridged critical knowledge gaps for patients.

Across the semantic networks of the top 10 OLs’ posts and their associated replies (Figures 3-12), informational support consistently predominates, manifesting as clusters centered on treatment protocols, diagnostic guidance, and clinical expertise. Emotional support, while present, remains secondary. This pattern aligns with the platform’s asynchronous, text-based nature, which favors knowledge dissemination over affective exchange, and highlights the expert-driven character of influence in TB-related OHCs.

## Discussion

Despite the growing importance of OHCs, empirical research on TB-related OHCs remains limited, leaving a significant gap in understanding the nature of social support available to affected individuals amid increasing internet access in China. This study helps bridge this gap by examining the TB forum on Baidu Post Bar, a prominent TB-related online community in China. Focusing on its subforum of administrator-recommended posts and associated replies, we applied content analysis, social network analysis, and semantic analysis to a longitudinal dataset spanning 18 years from the TB forum’s inception in 2004 to 2021.

### Principal Findings

The literature suggests OHCs as an important source of social support for health issues [12,13], which is confirmed by this study in the context of TB. In the leading TB online forum in China examined in this study, 67.5% (296/438) of the administrator-recommended posts contained social support. Consistent with previous studies on OHCs [25-27], informational and emotional support were the most common types. Moreover, there were more posts providing support than those seeking it, fostering a supportive online environment. Out of the 296 posts containing social support, informational support was the most prevalent, appearing in 150 posts—129 providing it and 21 seeking it. Emotional support was also significant, present in 136 posts, with 77 providing it and 59 seeking it. While access to traditional social support programs remains a major challenge for people affected by TB and their households [8], increased internet access has provided a channel for social support, which could be further leveraged for effective digital health interventions.

An examination of the 3 most common themes—treatment experience, nontreatment sharing, and disease knowledge—revealed distinct patterns of social support. Among the 129 posts on treatment experience, 115 contained social support, primarily seeking emotional support (47 posts). Of the 113 nontreatment sharing posts, 41 included social support, primarily providing emotional support (28 posts). For the 75 posts about disease knowledge, providing informational support was overwhelmingly dominant, appearing in 74 posts.

The social network analysis identified 10 leading OLs based on a Borda ranking method, which reflects overall influence derived from 4 centrality measures and user tenure as a temporal factor. While centrality measures capture users’ positions and interaction patterns within the network, user tenure reflects the longevity of participation and is interpreted in this study as a complementary, rather than controlling, dimension of influence.

Specifically, the first-ranked OL not only achieved the highest combined in-degree and out-degree centrality but also topped each individually, indicating the highest levels of replies received and sent. The second-ranked OL placed second in combined centrality. Among the 438 posts, the first OL contributed 71 (16.2%) posts, and the second contributed 62 (14.2%) posts, accounting for a combined 30.4% (133/438) of all posts.

Consistent with previous research identifying OLs in OHCs primarily as patients [12], the top-ranked OL in this study was a former patient with TB. This individual’s experiences of completing treatment and achieving full recovery likely provided substantial disease knowledge and a positive outlook, both of which have been shown to facilitate opinion leadership in OHCs [42]. The second-ranked OL was a pulmonary TB doctor with professional expertise. In addition, the first OL’s role as the TB forum’s administrator and the second OL’s role as the forum’s initial administrator highlight the significance of administrative positions in shaping influence through higher posting volume and denser reply-based interactions.

Importantly, tenure was not used to normalize or adjust the centrality measures. Rather, it was included as a distinct indicator in the Borda aggregation to reflect cumulative presence in the community. Accordingly, the Borda ranking should be interpreted as capturing overall influence, which may derive from both structural embeddedness and long-term participation. Structural centrality and tenure are therefore analytically distinguished in this study, and high tenure alone does not imply opinion leadership in the absence of strong network centrality.

Finally, the semantic network analysis uncovered the topic clusters in each OL’s posts and their associated user replies. Across these semantic networks, informational support was more dominant than emotional support. Between the 2 highest-ranked OLs who served as administrators of the TB forum, their semantic networks shared common themes, including treatment information for TB and pulmonary TB, as well as the importance of consulting doctors. However, their themes diverged in scope. As a former patient with TB, the first OL addressed a broader range of topics and provided more emotional support, as reflected by 1 theme conveying well wishes for recovery and another theme containing holiday greetings. The first OL’s most-replied-to post fostered social support within the forum by urging patients with TB who had completed medication treatment to respond and encourage newly diagnosed patients with TB. As a pulmonary TB doctor, the second OL provided more informational support, drawing on professional expertise. One theme focused on clinical symptoms and pathology, using more medically specific language. In the second OL’s most-replied-to post, titled “Ask the Doctor,” professional information and advice were shared.

These findings demonstrate that OLs predominantly provide informational and emotional support while anchoring the dominant topical clusters. This pattern offers empirical validation for an adapted 2-step flow model within OHCs, wherein influential users act as gatekeepers, selectively filtering and amplifying health information. The triangulation of methods further reveals interdependent mechanisms: structural positions (captured through social network analysis) systematically shape discursive content (manifest in semantic clusters) and support provision (coded via content analysis). Collectively, these insights yield a multilevel framework of OHC functioning that transcends descriptive enumeration, theorizing how relational architecture mediates both knowledge dissemination and affective sustenance in stigmatized illness contexts.

### Limitations

This study has several limitations that warrant consideration. First, our analysis was confined to the subforum that features administrator-recommended posts, which are widely regarded by forum users as high-quality content and generate dense interaction patterns. While this focus ensured a cohesive and analytically tractable dataset suitable for network analysis, it excluded low-engagement threads from the broader forum. Importantly, restricting the network to threads curated by administrators may introduce a circular validation effect, whereby administrators are more likely to appear structurally central due to their role in initiating or curating these discussions.

We acknowledge that a sensitivity analysis—such as reconstructing the network after excluding administrator-authored initial posts to examine whether administrators remain central based solely on reply-based interactions—would provide a more rigorous test of this potential bias. However, such analyses were not conducted in this study. Consequently, the identified opinion leadership, particularly among administrators, should be interpreted as conditional on this curated subforum context rather than as a definitive representation of influence across the entire TB forum. Future research should address this limitation by incorporating the full forum dataset and conducting sensitivity analyses to assess the robustness of centrality findings under alternative sampling and network construction strategies.

Second, the cross-sectional aggregation of data across 2004-2021 obscures temporal evolution. Longitudinal disaggregation—for example, annual or periodic analysis of support types, OL prominence, and topical shifts—could illuminate how social support and leadership structures adapted to changing platform vitality and external health events.

Finally, Baidu Post Bar’s declining usage in recent years amid competition from newer platforms limits the generalizability of findings to the contemporary Chinese OHC landscapes. Comparative studies of TB-related online communities on emerging social media platforms would enrich the literature and reveal platform-specific mechanisms of support provision and influence diffusion.

### Conclusions

This study demonstrates that the TB forum on Baidu Post Bar served as an important source of social support for people affected by TB in China, fostering an environment rich in both informational and emotional resources. Of the 438 analyzed posts, 67.5% (296/438) contained social support, with informational support appearing in 150 posts and emotional support in 136. Thematic distribution revealed distinct patterns: disease knowledge posts overwhelmingly provided informational support (74/75), while treatment experience posts most frequently sought emotional support (47/129), and nontreatment sharing posts most commonly offered emotional support (28/113).

Using a Borda ranking method that integrates 4 centrality measures and user tenure, we identified 10 OLs. Semantic network analysis of each OL’s posts and their associated user replies revealed that informational support predominated over emotional support. The 2 highest-ranked OLs—a former patient with TB and a pulmonary TB doctor—exerted the strongest influence through reply interactions. While both emphasized TB treatment and the importance of medical consultation, the patient OL covered a wider range of topics and provided more emotional support, whereas the doctor OL focused predominantly on authoritative informational content.

These findings suggest that OLs play a central role in filtering and amplifying health information, consistent with an adapted 2-step flow model in OHCs. The integration of content analysis, social network analysis, and semantic network analysis not only maps the structure of support provision but also reveals how structural positions (centrality), discursive content (topical clusters), and support types interact to sustain community functioning.

## Abbreviations

OHC: online health community
OL: opinion leader
TB: tuberculosis
